# The nuclear interactome of DYRK1A reveals a functional role in DNA damage repair

**DOI:** 10.1038/s41598-019-42990-5

**Published:** 2019-04-25

**Authors:** Steven E. Guard, Zachary C. Poss, Christopher C. Ebmeier, Maria Pagratis, Helen Simpson, Dylan J. Taatjes, William M. Old

**Affiliations:** 1 Department of Molecular, Cellular and Developmental Biology, University of Colorado, Boulder, CO USA; 2 Department of Biochemistry, University of Colorado, Boulder, CO USA; 3 Linda Crnic Institute for Down Syndrome, University of Colorado School of Medicine, Aurora, CO USA

**Keywords:** Protein-protein interaction networks, DNA damage response

## Abstract

The chromosome 21 encoded protein kinase DYRK1A is essential for normal human development. Mutations in DYRK1A underlie a spectrum of human developmental disorders, and increased dosage in trisomy 21 is implicated in Down syndrome related pathologies. DYRK1A regulates a diverse array of cellular processes through physical interactions with substrates and binding partners in various subcellular compartments. Despite recent large-scale protein-protein interaction profiling efforts, DYRK1A interactions specific to different subcellular compartments remain largely unknown, impeding progress toward understanding emerging roles for this kinase. Here, we used immunoaffinity purification and quantitative mass spectrometry to identify nuclear interaction partners of endogenous DYRK1A. This interactome was enriched in DNA damage repair factors, transcriptional elongation factors and E3 ubiquitin ligases. We validated an interaction with RNF169, a factor that promotes homology directed repair upon DNA damage, and found that DYRK1A expression and kinase activity are required for maintenance of 53BP1 expression and subsequent recruitment to DNA damage loci. Further, DYRK1A knock out conferred resistance to ionizing radiation in colony formation assays, suggesting that DYRK1A expression decreases cell survival efficiency in response to DNA damage and points to a tumor suppressive role for this kinase.

## Introduction

In humans, haploinsufficiency of DYRK1A is associated with a neurodevelopmental disorder (OMIM 614104) characterized by intellectual disability, microcephaly, and autism^1,2^. Conversely, DYRK1A overexpression is implicated in some of the more severe clinical manifestations of trisomy 21 (Down syndrome)^3–5^, including childhood acute megakaryoblastic leukemia^6^, disrupted neurodevelopment^7,8^, early onset Alzheimer’s disease^9,10^, and decreased risk of solid tumors^11,12^. Together, the evidence points to an acute sensitivity during human development to alterations of DYRK1A copy number, acting through mechanisms that are largely unknown.

Although most of the *de novo* DYRK1A mutations associated with human neurodevelopmental phenotypes have been shown to disrupt kinase activity *in vitro*^13,14^, a number of clinically relevant non-synonymous mutations outside of the kinase domain failed to disrupt wild-type activity, pointing to kinase-activity independent functions of DYRK1A during brain development^14^. In contrast to many protein kinases that are activated through reversible phosphorylation events, DYRK1A activity is constitutively activated by a co-translational autophosphorylation event^15,16^, and is thought to be regulated through subcellular compartmentalization^17^, transcriptional control^18^, and protein-protein interactions^19^. Kinase activity-independent roles have been reported for DYRK1A in regulating Arip4 transcriptional activation^20^, and recruitment to serum-responsive promoter elements^21^, suggesting that its functions extend beyond phosphorylation to non-catalytic mechanisms such as scaffolding and protein-DNA interactions, as observed for other protein kinases^22^.

While cytosolic DYRK1A has better known roles in regulating the cell cycle^8^ and cytoskeletal dynamics^23^, its functions within the nucleus are more enigmatic^24^. DYRK1A contains a bipartite nuclear localization signal within its kinase domain that is required for nuclear localization, and a C-terminal poly-histidine tract that is required for nuclear speckle localization^25^ and phase-separation with RNA polymerase II^24^. Phosphorylation of various SRSF splicing factors by DYRK1A has been shown to regulate alternative splicing of Tau^26^. DYRK1A has also been reported to regulate transcription machinery through kinase dependent and independent interactions with RNA polymerase II C-terminal domain^21,24^. Despite the accumulating evidence linking DYRK1A to important cellular processes within the nucleus, many of the molecular interactions underlying these functions are not completely known.

Most of the known DYRK1A interactions were discovered in low-throughput reciprocal IP-western studies^27^ and large-scale interactome studies using affinity-purification mass spectrometry (AP-MS) analysis^28–30^. As a methodology, AP-MS has enabled large-scale interrogation of the human protein-protein interactome, providing insights into function for the large fraction of the proteome that has no functional annotation^31^. However, the ectopic expression systems commonly employed lack regulatory elements and local chromatin environments required to recapitulate endogenous expression levels. Consequently, stoichiometric balances for multiprotein complexes and pathways can be disrupted, particularly for dosage-sensitive genes^32–34^. Non-physiological overexpression of DYRK1A has been shown to alter its subcellular distribution^35^, confounding the interpretation of DYRK1A interaction studies that employ ectopic expression.

To circumvent these issues and identify DYRK1A protein interactions within the nucleus, we performed mass spectrometry analysis of immunoaffinity-purified endogenous DYRK1A from HeLa nuclear extracts. The resulting interactome revealed many previously unreported interactions, representing a significant increase in the number of known DYRK1A interaction partners. We identified central regulators of transcription and DNA damage repair, including RNF169, members of the BRCA1-A complex, and four subunits of the super elongation complex, consistent with emerging evidence for DYRK1A-dependent regulation of these processes^21^. We found that knockout of DYRK1A or treatment with DYRK1A inhibitors antagonizes DNA double strand break repair kinetics, and that DYRK1A protein expression decreased following induction of DNA double strand breaks by IR. DYRK1A expression was also found to be required for maintenance of 53BP1 expression in unirradiated HeLa cells. Finally, we found that CRISPR/Cas9 knockout of DYRK1A in HeLa cells conferred resistance to ionizing radiation (IR). Our results reveal a new role for DYRK1A in DNA damage repair, with potential implications for radioresistance and tumor suppressive mechanisms in cancer.

## Results

### Nuclear interactome of endogenous DYRK1A

**Figure 1:**
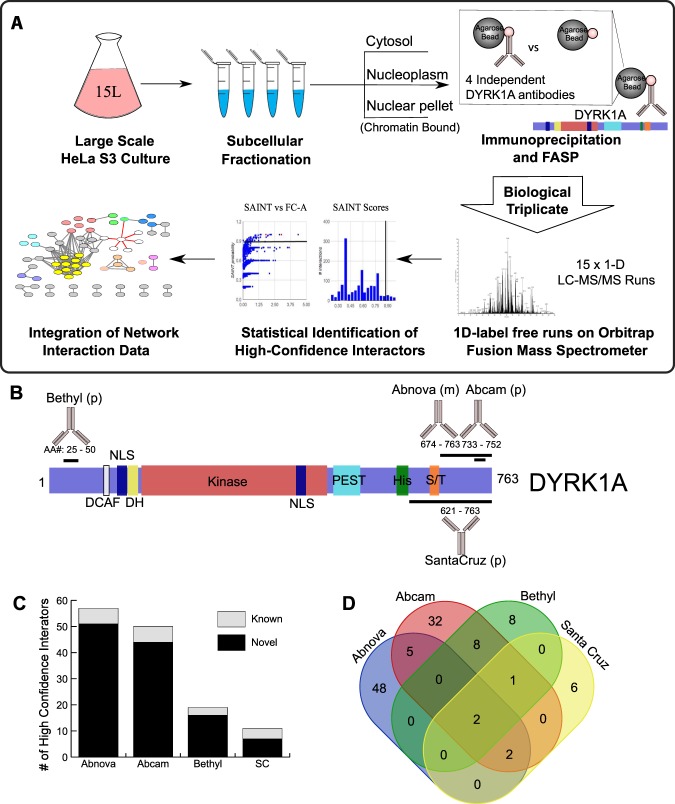
Workflow and strategy for generation of endogenous DYRK1A interactome. **(A)** Schematic overview of fractionation and IPMS strategy. HeLa S3 cells were grown in 6 L flasks before harvesting at 1 million cells/ml. Large scale fractionation of cell pellets generated protein lysates representing a cytosolic, nuclear and chromatin bound fractions. Subsequent immunoprecipitations were done in triplicate prior to sample preparation for mass spectrometry identification. **(B)** Domain arrangement of DYRK1A and regions containing antibody epitope as reported by manufacturers. NLS, nuclear localization signal; DH, DYRK homology box; PEST, proline glutamic acid serine and threonine rich sequence; His, polyhistidine stretch; S/T serine and threonine rich region. Epitopes recognized by each antibody used for IP experiments fall within the designated amino acid region and represented by black bar. (p) rabbit polyclonal; (m) mouse monoclonal. **(C)** Number of novel and literature reported HCI’s by antibody. (Abnova n = 3; Abcam n = 3; Bethyl n = 3; SC: Santa Cruz n = 3) FC-A > 3; SAINT > 0.7. **(D)** Overlap between HCI’s of each set of immunoprecipitations by antibody.

To identify interaction partners of endogenous, nuclear-localized DYRK1A, we immuno-purified DYRK1A in triplicate from a large-scale preparation of HeLa cell nuclear extracts, using four different commercial antibodies, followed by quantification with label-free mass spectrometry (IP-MS) (Fig. 1A). The four DYRK1A antibodies recognize different epitopes in the N-terminal and C-terminal regions of human DYRK1A (Fig. 1B; Suppl. Table S1). This strategy ensures maximal coverage of interaction partners in the event that an antigenic surface overlaps with a protein interaction interface and disrupts capture of endogenous prey interactions. Immunoprecipitated proteins were digested into tryptic peptides using filter aided sample prep (FASP)^36^, and analyzed by 1D liquid chromatography tandem mass spectrometry using Orbitrap Fusion instrumentation^37^.

In affinity proteomics experiments, quantification of bait-prey interactions relative to a control is critical for distinguishing true interacting proteins from non-specific background. This is particularly important for affinity purifications from complex lysates, in which non-specific interactions predominate as a function of total protein^38^. To distinguish true DYRK1A-interacting proteins from non-specific background, we used beads-only controls and the CRAPome analysis tool^38^. This approach calculates two measures: a posterior probability of true interaction using the SAINT algorithm^39^, and a fold-change enrichment, FC-A, which estimates an enrichment over internal user controls. High confidence interactions (HCIs) were defined as proteins with an FC-A of 3.0 or greater and a SAINT probability of 0.7 or greater. Our analysis revealed a total of 105 HCIs, 97 of which have not been reported as DYRK1A interacting proteins (Fig. 1C,D; Suppl. Table S2). For each antibody, DYRK1A was identified within the four most highly enriched proteins, demonstrating high-specificity toward DYRK1A from nuclear extracts. A core set of 5 HCIs were shared by three or more antibodies, which included the known DYRK1A interacting proteins DCAF7, GLCCI1, RNF169, TROAP, and FAM117B (Fig. 1D)^30,40^. While the Abnova and Abcam antibodies resulted in the identification of several-fold more interactions than the SantaCruz and Bethyl antibodies, most represented unique, novel interactions (Fig. 1C,D). This suggests that these antibodies recognize distinct DYRK1A sub-complexes, potentially due to epitope overlap within a protein-protein interaction surface.

**Figure 2:**
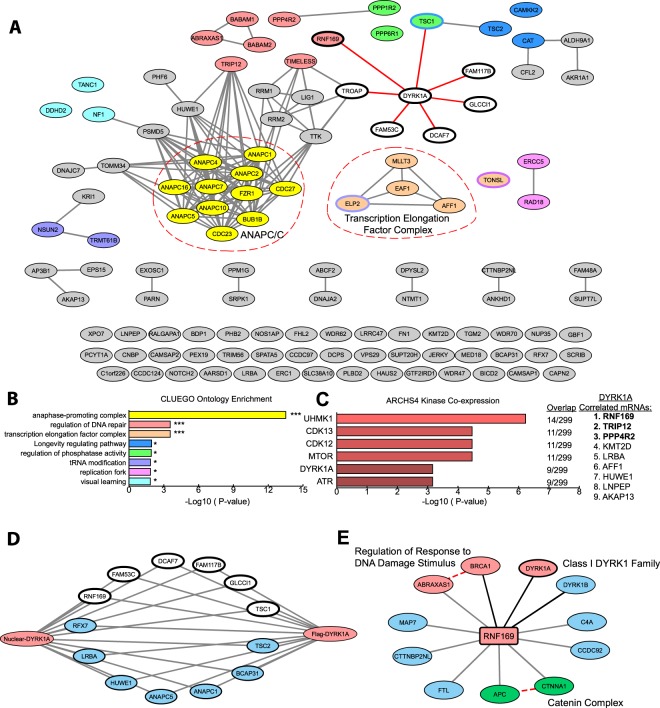
Network analysis of protein-protein interactions (PPIs) from nuclear DYRK1A interactome. (**A)** STRING-DB network of high significance PPIs from nuclear DYRK1A interactome. Nodes in network represent proteins found in a shared HCI list between antibodies. Total Nodes: 106. HCI cutoffs: FC-A > 3, SAINT > 0.7. Grey edges denote STRING-DB evidence of 0.400 for interactions between HCIs. Red edges denote confirmed BioGRID interactions. Color of node correlates to GO ontologies outlined in (**B**). (**B**) ClueGO ontology enrichments from total HeLa nuclear DYRK1A interactome (Fisher exact test: *p < 0.05; **p < 0.01; ***p < 0.001). **(C)** Enrichment of HCIs against the ARCHS4 RNA-seq database for kinase co-expression. (Lists of top 300 most correlated mRNAs for each kinase are generated through Enrichr.) Bold: GO:2000779 Regulation of double-strand break repair (Fisher exact test: adj. p = 0.00004). **(D)** Protein-protein interaction network overlap between nuclear interactome from (**A**), and 3x FLAG_FL_DYRK1A co-purification from HEK293 whole cell lysate; proteins were identified through label free MS/MS 1D runs. (FC-B > 3.0; n = 2). White nodes/bold outline denotes confirmed BioGRID interactions for DYRK1A. **(E)** RNF169 nuclear interactome (FC-A > 3; SAINT > 0.7) n = 2. Grey edges denote high confidence interactions; Red edges: STRING-DB evidence 0.400. Regulation of response to DNA damage stimulus p = 0.01; Catenin complex p = 0.007.

To gain insight into biological functions associated with the DYRK1A HCIs, we performed functional enrichment and STRING network analysis. HCIs identified in each of the four sets of immunoprecipitations were combined and mapped onto a STRING protein-protein interaction network (Fig. 2A). Functional enrichment analysis using ClueGO^41^ revealed several distinct clusters of functionally related proteins and multiprotein complexes involved in transcription and DNA damage repair (Fig. 2B). For example, we identified three of five subunits from the BRCA1-A complex, which regulates repair of radiation induced DNA damage^42,43^, as well as TRIP12, RAD18 and ERCC5 (Fig. 2A). Interestingly, haploinsufficiency of TRIP12 has been reported to cause intellectual disability^44^, which is a clinical presentation shared with DYRK1A haploinsufficiency.

The most significantly enriched term from the ClueGO analysis is related to the anaphase-promoting complex (APC/C) (Fig. 2B), for which we identified 10 of the 15 core subunits (Fig. 2A,B; Suppl. Fig. S1A). APC/C is a 1.2 MDa E3 ubiquitin ligase complex that controls mitotic progression by regulating the activity of cyclin-dependent kinases^45^ and has been recently implicated in regulation of DNA damage repair pathway choice^46^. Substrate specificity of APC/C is determined by mutually exclusive association with the adaptor proteins FZR1 (Cdh1) and CDC20, which bind APC/C in a cell cycle dependent manner^47^. Interestingly, in our list of DYRK1A HCIs, we found FZR1, but not CDC20, suggesting that DYRK1A could be preferentially interacting with the APC/C complex between late mitosis and G1 when FZR1 is the primary cofactor^48^.

mRNA expression levels for physically interacting proteins have been postulated to co-evolve, to explain unexpectedly high pairwise expression correlation^49^. Given a suitably large compendium of expression data, these correlation patterns can be used to infer novel protein-protein interactions via co-expression analysis^50^. Using this principle, we asked whether enrichment of co-regulated mRNAs for proteins in our interactome might reveal stable interactions within our DYRK1A HCIs. We therefore tested for significant overlap of proteins in our DYRK1A interactome with ARCHS4 kinase co-expression modules using Enrichr^51^. As expected, we found that the module of DYRK1A co-regulated mRNAs showed significant overlap with our DYRK1A HCIs. Examination of these mRNAs revealed that many were involved in DNA damage (Fig. 2C), suggesting that DYRK1A may be involved in DNA damage repair, potentially through association with protein complexes that are recruited to DNA double-strand breaks (DSBs).

### Interaction with RNF169 implicates DYRK1A in DNA damage repair

The DNA damage protein RNF169 was consistently represented with the highest combination of SAINT scores and FC-A values across every replicate for all four DYRK1A antibody data sets in this study (Suppl. Fig. S1B–E). RNF169 has emerged as an important factor influencing DNA repair pathway outcome upon DSB formation, and has been shown to promote high-fidelity homologous recombination (HR) repair^52,53^ and single-strand annealing repair^54^. RNF169 functions to compete with a crucial non-homologous end-joining (NHEJ) driver, 53BP1, for binding of H2A ubiquitin marks near the sites of damage^55,56^. RNF169 binding thereby antagonizes the early kinetics of NHEJ in favor of slower HR repair^54,57,58^.

Physical association between RNF169 and DYRK1A was demonstrated using two orthogonal biochemical approaches. First, we transfected HEK293T cells with a FLAG-DYRK1A construct and quantified the proteins that co-purified with DYRK1A from whole cell lysate using mass spectrometry. We observed RNF169 and APC/C subunits among the many proteins that overlap between these two cell-type specific interaction networks (Fig. 2D). This is consistent with previous AP-MS data sets that found DYRK1A:RNF169 interactions from HEK293T and SH-SY5Y cells in whole cell lysates (Suppl. Fig. 1G)^30,59^. Second, we performed reciprocal IP-MS of endogenous RNF169 immunopurified from HeLa nuclear extracts. Consistent with the DYRK1A IP-MS data, DYRK1A was identified in RNF169 IPs, in addition to DYRK1B, a class I DYRK kinase. Interestingly, DYRK1B was not found in the DYRK1A IP-MS data, suggesting mutually exclusive binding of DYRK1A and DYRK1B to a common interaction surface of RNF169. Among the RNF169 HCIs were subunits of the BRCA1-A complex: ABRAXAS1 and BRCA1 (Fig. 2E). This complex was well represented in the DYRK1A interactome by proteins ABRAXAS1, BABAM1 (also known as NBA1 and MERIT40) and BABAM2 (also known as BRE and BRCC45) (Fig. 2A). The shared interaction of both DYRK1A and RNF169 with BRCA1-A subunits is interesting in light of the requirement for this complex in efficient homology directed repair of DSBs^42,60–62^.

### DYRK1A levels influence DNA DSB repair protein recruitment

**Figure 3:**
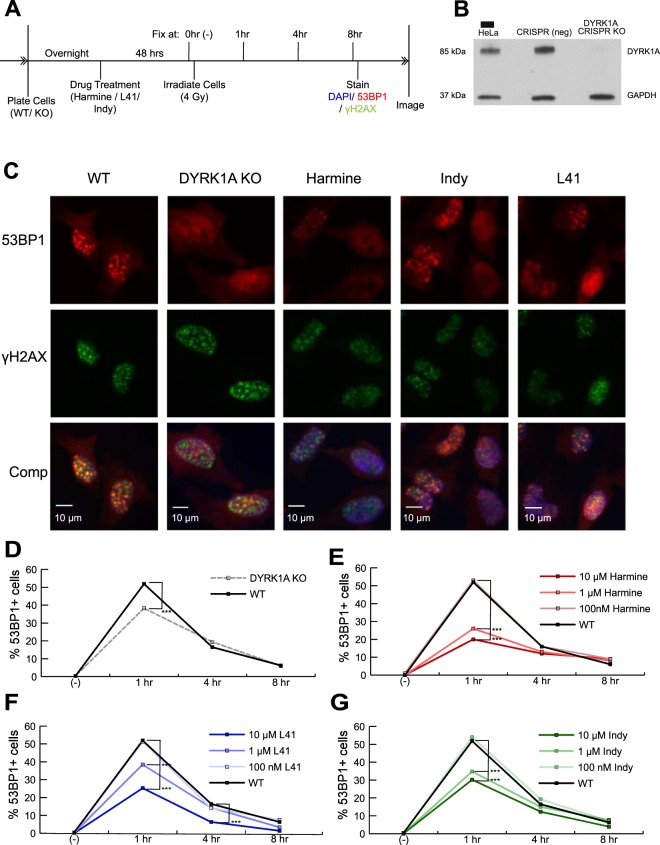
DYRK1A expression and kinase activity are necessary for efficient 53BP1 recruitment to DNA double-strand break sites. **(A)** Experimental timeline for 53BP1 foci quantitation in response to DYRK1A manipulation and IR. Cells were treated with drug 48 hours prior to treatment with 4 Gy of IR. Cells were fixed immediately before (0 hrs) or 1, 4 and 8 hrs following IR treatment. **(B)** Western blot confirmation of DYRK1A CRISPR knockout in HeLa cells. DYRK1A and GAPDH loading control bands were cropped from portions of the same blot (Full molecular weight range seen in Suppl. Fig. S3E). **(C)** Representative immunofluorescent images of γH2AX and 53BP1 staining in fixed HeLa cells. Cells were fixed in 96-well plates. Four frames per well were imaged for each well. Four wells per condition per time point were plated and quantified using the Focinator R package^79^. N ≈ 400–1000 cells per condition. Blue: Hoechst; Green: γH2AX; Red: 53BP1. 53BP1 + cells ≥ 10 foci/cell. (53BP1 noise cut off: 15; γH2AX noise cut off 20). **(D)** DYRK1A KO HeLa cells: Proportion of 53BP1 + cells over time following 4 Gy of IR (***p < 0.001). **(E**–**G)** WT HeLa cells were treated with Harmine, L41 or INDY for 48 hours prior to irradiation: Proportion of 53BP1 + cells over time following 4 Gy of IR (***p < 0.001).

The interaction between DYRK1A and RNF169, among other regulators of DNA double strand break repair, suggested that DYRK1A could play a role in regulating DNA damage repair. To test this idea, we examined the effect of DYRK1A knockout (KO) and pharmacological inhibition of DYRK1A activity with harmine on the localization of 53BP1 to sites of DNA double strand breaks induced by IR (Fig. 3A). 53BP1 is a driver of non-homologous end joining and is recruited to double-strand DNA lesions within 15 minutes of IR induced damage^58^. WT and DYRK1A KO HeLa cells were pre-treated with harmine and irradiated at 4 Gy, a dose selected to induce damage while minimizing widespread apoptosis. Following induction of double strand breaks by IR, cells were fixed at 1, 4 and 8 hours and stained for γH2AX, 53BP1 and Hoechst to visualize and quantify the formation and resolution of IR induced foci. We found that 53BP1 foci formation at sites of damage maximized one-hour post IR and that these foci resolve in most of the population by 8 hours (Suppl. Fig. S2A). We observed that unirradiated HeLa cells typically contain basal levels of γH2AX foci and singular, large 53BP1 foci characteristic of stalled replication forks and inherited DNA damage lesions (Suppl. Fig. S2A)^63^. Consequently, to quantify cells containing only IR-induced foci, we defined 53BP1 positive cells as containing greater than 10 foci per nucleus, as described previously^53^.

Relative to the parental line, DYRK1A KO cells exhibited a decreased proportion of 53BP1 + cells at one hour post IR that resolved to wild-type levels by 8 hours, consistent with impaired recruitment of 53BP1 to IR induced foci (Fig. 3C,D). Consistent with knockout of DYRK1A, pre-treatment with harmine led to a significant, dose-dependent reduction of 53BP1 recruitment (Fig. 3E), suggesting that DYRK1A’s kinase activity is required. Treatment with the structurally unrelated DYRK1A inhibitors L41^64^ and INDY^65^ phenocopied harmine treatment and DYRK1A KO cells, showing impaired 53BP1 foci formation in a dose dependent manner (Fig. 3F,G).

Surprisingly, when DYRK1A KO cells were treated with harmine prior to IR, 53BP1 foci formation was further reduced (Suppl. Fig. 2B), pointing to an off-target effect of harmine. While minimizing most off-target kinases with the panel of drugs used here, the class I DYRK family member, DYRK1B, is inhibited by INDY and L41 at approximately the same concentration as DYRK1A, and is inhibited by harmine with an IC50 2–3 times higher than that for DYRK1A^64,66,67^. As both DYRK1A and DYRK1B were identified as RNF169 interacting proteins (Fig. 2E), we speculate that the catalytic activity of these kinases could be acting in a semi-redundant manner to regulate 53BP1.

### DYRK1A is required for maintaining basal 53BP1 expression

**Figure 4:**
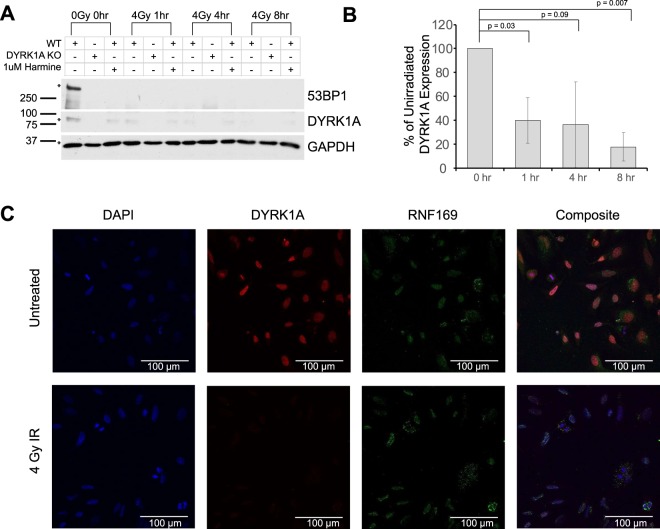
DYRK1A expression and kinase activity are necessary for maintaining basal 53BP1 expression. (**A)** Immunoblot for 53BP1, DYRK1A and GAPDH expression in either WT, DYRK1A KO or 1 µM harmine treated WT cells at 0, 1, 4, and 8 hrs following 4 Gy of IR. Bands were cropped from portions of the same blot. (Full molecular weight range seen in Suppl. Fig. S3C). **(B)** Quantification of DYRK1A expression from three independent experiments outlined in 4a (student’s t-test). **(C)** Representative immunofluorescent images of DAPI, DYRK1A and RNF169 staining 1 hour post- 4 Gy of Irradiation.

In cells with no discernable 53BP1 foci at sites of DNA damage, a diffuse 53BP1 signal was localized to the nucleoplasm (Fig. 3C). Together with reported observations that all non-chromatin bound 53BP1 protein is degraded upon induction of DNA double strand breaks^68^, we speculated that 53BP1 protein expression may be altered in a DYRK1A-dependent manner, leading to impaired 53BP1 recruitment upon IR-induced DNA damage. We first examined 53BP1 expression in unirradiated WT, DYRK1A KO and harmine treated HeLa cells. Unexpectedly, 53BP1 protein expression decreased below detectable levels in DYRK1A KO and harmine treated cells prior to induction of DNA damage (Fig. 4A). Loss of 53BP1 protein expression in unirradiated cells in response to harmine treatment was found to be dose dependent and occurred as early as 3 hours after drug treatment (Suppl. Fig. 4A). As expected, 53BP1 expression in WT cells dropped to undetectable levels at 1 hr post-IR and remained undetectable at 8 hours. Because DYRK1A expression and kinase activity was required for 53BP1 protein expression in unirradiated cells, we next asked whether DYRK1A expression was altered in response to IR-induced damage.

In response to IR, DYRK1A expression decreased slowly over 8 hours in WT cells (Fig. 4A,B; Suppl. Fig. 3D). To confirm this loss of DYRK1A expression, we examined the localization of DYRK1A and RNF169 in WT Hela cells during DNA double strand break repair at one-hour post IR. Under basal conditions, DYRK1A localized diffusely in the nucleoplasm with small puncta characteristic of known recruitment to nuclear speckles^25^, but upon IR, the majority of this signal was lost (Fig. 4C). Additionally, DYRK1A and RNF169 staining patterns did not show colocalization at sites of DNA damage, suggesting that DYRK1A:RNF169 interactions are not concentrated at the sites of IR-induced damage (Fig. 4C). Taken together, these results indicate that DYRK1A is required for maintenance of 53BP1 expression and subsequent recruitment to DSBs. Interestingly, this contrasts with the reported role for RNF169 in antagonizing 53BP1 recruitment to promote HR and single-strand annealing repair^52,54^.

### DYRK1A knock-out confers resistance to ionizing radiation

**Figure 5:**
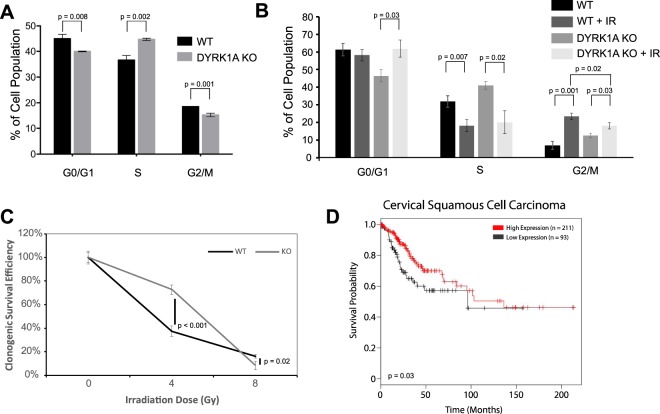
Knockout of DYRK1A promotes radioresistance and cell survival. **(A)** Quantification of proliferating HeLa cells by cell cycle phase: flow cytometry analysis of propidium iodide staining and BrdU incorporation; 10 µM BrdU was pulsed 60 minutes prior to harvest. n = 3 (student’s t-test). **(B)** Quantification of HeLa cells by cell cycle phase 18 hours following 4 Gy of IR: flow cytometry analysis was done using propidium iodide staining of either WT or DYRK1A KO HeLa cells. n = 3 (student’s t-test). **(C)** Results of clonogenic survival assay show increased radioresistance of DYRK1A KO cells above WT survival following 4 Gy of IR. Cell colonies were counted using clono-counter java package 12 days following initial radiation. **(D)** Kaplan-Meier survival analysis of cervical squamous cell carcinoma tumors generated through KM plotter^74,75^. Patients with high DYRK1A expression in their tumors had increased survival probability over those with low DYRK1A expression.

Pathway choice in DNA double strand break repair is dependent on cell cycle phase, with HDR peaking in S-phase, and NHEJ peaking in G1 and G2 phases^69^. We therefore compared differences in cell cycle distribution between WT and DYRK1A KO cells under basal conditions and in response to double strand breaks. Quantification of cell cycle phase by flow cytometry showed that the fraction of cells in S-phase was modestly increased in DYRK1A KO cells relative to the control line (Fig. 5A), consistent with previous studies^70^. However, 18 hours after IR treatment, when cells are expected to be maximally arrested^71^, the proportion of cells in G1/G0, and S phase for DYRK1A KO cells normalized to WT levels (Fig. 5B). This suggests that while DYRK1A KO cells proliferate at a higher rate, cell cycle checkpoints remain intact to facilitate timely DNA damage repair.

Evidence thus far is consistent with a function for DYRK1A in DSB repair through the regulation of 53BP1, and suggests that DYRK1A expression may affect cell survival in response to IR. To test this idea, we compared clonogenic survival for DYRK1A KO and WT cells following treatment with IR. We found that DYRK1A KO cells showed a dramatically increased survival rate over 10 days relative to WT cells, after exposure to 4 Gy of IR (Fig. 5C). Thus, loss of DYRK1A conferred radioresistance in this colony formation assay, suggesting that DYRK1A expression decreases cell survival in response to DNA damage. This result is consistent with a previous report of DYRK1A-dependent radiosensitivity in colorectal adenocarcinoma cells^72^. As radioresistance in cancer is correlated with poor prognosis^73^, we performed Kaplan-Meier analysis of curated RNA-seq datasets representing 20 different cancer types^74,75^ to determine whether DYRK1A expression correlated with patient survival across a variety of cancers. We found that high DYRK1A mRNA expression was correlated with increased patient survival in cervical squamous cell carcinoma, among several other tumor types representing a wide range of cancers (Fig. 5D; Suppl. Fig. 4A). Collectively, this data indicates that DYRK1A activity and expression is required for efficient repair of IR-induced DNA double strand breaks, and that changes in its expression levels influence cell survival in response to genomic insult.

## Discussion

In this study, we performed a deep proteomic analysis of DYRK1A-associated nuclear proteins by mass spectrometry analysis of immunopurified endogenous DYRK1A. We identified 105 high-confidence DYRK1A interactions, only 8 of which that have been described previously, underscoring how current knowledge of DYRK1A interactions is incomplete. This issue is not limited to DYRK1A; a recent AP-MS interactome study of all human CMGC kinases found that more than 75% of the discovered interactions were previously unknown^30^. Enrichment of DYRK1A from nuclear extracts in this study likely contributed to the large number of unique interactions compared with previous studies, which used whole cell lysates^30,59^. The nucleus contains approximately 15% of the total cellular protein in HeLa cells^76^, and accordingly, low abundance proteins from this compartment are likely to be under sampled relative to more abundant cytosolic interactions when using whole cell lysates. Although many of the interactions unique to this study appeared across cell lines and purification methods, further characterization of these interacting partners will be required to distinguish between primary and secondary interactions.

A small set of DYRK1A HCIs identified here have been independently identified by multiple studies: RNF169, FAM117B, GLCCI1, TSC1, DCAF7, TROAP, FAM53C and TRMT61B (Fig. 2A; Suppl. Fig. 1G). These represent a core set of conserved, high affinity interaction partners of DYRK1A, due to their consistent appearance across different cell types employed in previous affinity purification studies. Remarkably, four of these interactors, TROAP, GLCCI1, FAM53C and FAM117B, have very limited functional annotation. This highlights how our understanding of human DYRK1A function remains limited, nearly three decades since the discovery of Yak1p, the founding member of the DYRK family in budding yeast^77^.

We wondered whether any of the antibodies used in this study might illuminate other studies that used the same reagents. Interestingly, all SEC subunits identified as DYRK1A interactions were only detected in DYRK1A IPs using the Abcam antibody (ab69811) that recognizes a C-terminal DYRK1A sequence (Fig. 1B). The same Abcam and Abnova antibodies employed here were used previously in ChIP experiments to uncover DYRK1A’s transcriptional function^21^. In that study, Di Vona *et al*. reported that DYRK1A was recruited to chromatin in HeLa cells based on ChIP experiments using the Abcam antibody (ab69811), while the Abnova DYRK1A antibody prevented binding of DYRK1A to DNA^21^. Taken together, this supports the notion that these antibodies may enrich for a distinct subset of DYRK1A interactions due to differential occlusion of a binding interface, and that DYRK1A recruitment to chromatin could be driven by secondary interactions with elongation factors or other unknown proteins.

Our DYRK1A interactome illuminates new functions for this protein kinase in DNA damage repair. The DNA damage proteins identified as HCIs in this study clearly implicate DYRK1A in regulating aspects of DNA damage repair, most likely in the regulation of 53BP1 protein expression prior to its localization at sites of damage. We found that DYRK1A associates with RNF169, which promotes homology directed repair mechanisms and antagonizes 53BP1 foci recruitment^52,54^. We show that DYRK1A expression is necessary for efficient recruitment of 53BP1 to DNA damage foci and is also required for maintenance of 53BP1 levels under basal conditions. Interestingly, DYRK1A expression decreased following IR treatment in WT cells, a process that may be required for canonical IR-induced 53BP1 degradation and subsequent repair by NHEJ. How this mechanism relates to the effects of DYRK1A expression on radiosensitivity and the tumor suppressive signature revealed in the Kaplan-Meier analyses remain unclear. Our findings have broad therapeutic implications related to roles for DYRK1A as a tumor suppressor and mediator of radiosensitivity. We speculate that DYRK1A expression could be used to predict response to radiation therapy in specific cancers. Moving forward, it will be crucial to elucidate the details of this mechanism to better understand DYRK1A’s contribution to DNA damage repair and cancer susceptibilities.

## Materials and Methods

### Antibodies

The following antibodies were purchased commercially: mouse monoclonal antibodies against DYRK1A (Abnova Corporation, Taipei, Taiwan; H00001859-M01) and phospho-Histone H2A.X (Ser139) (Millipore, Burlington, MA, 05-636), rabbit polyclonal antibodies against DYRK1A (Abcam, Cambridge, UK; ab69811; Bethyl Laboratories, Montgomery, TX; A303-801A; and Santa Cruz Biotechnology, Dallas, TX; sc-28899) and 53BP1 (Abcam; ab21083).

### Cell culture

HeLa cells were obtained from ATCC and cultured in DMEM + Glutamax + 10% FBS with antibiotic. DYRK1A knockout in a HeLa cell line was established using a CRISPR-Cas9 gene engineering method. Cells were transfected with an RFP-tagged Cas9 plasmid and two BFP-tagged sgRNA-plasmids (Sanger lentiviral CRISPR vector U6-gRNA: PGK-puro-2A-tagBFP, Sigma) using lipofectamine 3000 (Invitrogen, Carlsbad, CA6). Both sgRNA-plasmids contained guides to DYRK1A exon 5 of either 5′ ATGATCGTGTGGAGCAAGAATGG 3′ (plasmid #1) or 5′ TAAAATAATAAAGAACAAGAAGG 3′ (plasmid #2). All plasmids were provided by Josh Molishree, manager of the functional genomics facility at Anschutz Medical Center, University of Colorado, Denver, CO. Transfected cells were FACS-sorted and RFP/BFP positive cells were grown as single-cell clones from a 96 well plate. Clones were screened for loss of DYRK1A protein expression through western blot, T7 assay and sequencing of targeted region.

### Chemicals and treatments

DNA damage was induced by exposing cells to 4 Gy of X-ray irradiation using a Faxitron Cabinet X-Ray System (Faxitron, Tucson, AZ). Harmine (SantaCruz), L41 (BioVision, San Francisco, CA), and INDY (Tocris, Bristol, UK) stock solutions were prepared at 10 mM in DMSO. Drugs were then diluted down to final concentrations of 100 nM, 1 µM and 10 µM accordingly per experiment.

### Preparation of HeLa nuclear extract and nuclear pellet

HeLa nuclear extract was prepared from isolated nuclei from approximately 1 billion HeLa S3 cells, as described (Dignam *et al*.)^78^. The insoluble pellet from the nuclear extract (i.e. the nuclear pellet) was solubilized with 100 mM HEPES pH 7.9, 2 mM MgCl2, 100 mM KCl, 20% (v/v) glycerol, protease inhibitors (0.25 mM PMSF, 1 mM Sodium metabisulfite, 1 mM Benzamidine, 1 mM DTT), phosphatase inhibitors (1 µM Microcystin LR (Enzo Lifesciences, Farmingdale, NY), 0.1 mM Sodium orthovanadate, 10 mM beta-glycerophosphate, 5 mM sodium fluoride, 1 mM sodium pyrophosphate (all Sigma)), and nucleases Benzonase (200 U/mL) and DNAse I (50 U/mL). The pellet was chopped, dounce homogenized 20 times and mixed overnight with a stir bar at 4 °C. The extract was cleared by centrifugation at 14,000 x g and aliquoted for storage at −80 °C.

### Sample preparation for mass spectrometry

Affinity purified samples were precipitated with the addition of 10% (w/v) porcine insulin (Sigma), 0.1% (w/v) sodium deoxycholate, and 20% (w/v) trichloroacetic acid at 4 °C. Precipitated protein was pelleted and washed two times with −20 °C acetone and air dried. Samples were prepared for mass spectrometry using a modified version of the FASP method^36^. Samples were solubilized in 4%(w/v) sodium dodecyl sulfate (SDS), 100 mM Tris pH 8.5, 10 mM TCEP, boiled and allowed to reduce for 20 min, followed by alkylation with 25 mM iodoacetamide for 30 minutes in the dark. The reduced and alkylated proteins were then transferred to a 30 kD MWCO Amicon Ultra (Millipore) ultrafiltration device and concentrated, washed three times with 8 M urea, 100 mM Tris pH 8.5, and again three times with 2 M urea, 100 mM Tris pH 8.5. One microgram endoprotease LysC (Wako, Osaka, Japan) was added and incubated for 3 hrs rocking at ambient temperature, followed by 1 µg trypsin (Promega, Madison, WI), rocking overnight at ambient temperature. Tryptic peptides were collected by centrifugation and desalted using Pierce C-18 spin columns (Thermo Fisher Scientific) and stored dry at −80 °C.

### Mass spectrometry analysis

Samples were suspended in 3% (v/v) acetonitrile, 0.1% (v/v) trifluoroacetic acid and direct injected onto a 1.7 µm, 130 Å C18, 75 µm X 250 mm M-class column (Waters), with a Waters M-class UPLC. Tryptic peptides were gradient eluted at 300 nL/minute, from 3% acetonitrile to 20% acetonitrile in 100 minutes into an Orbitrap Fusion mass spectrometer (Thermo Scientific). Precursor mass spectrums (MS1) were acquired at 120,000 resolution (FWHM) from 380–1500 m/z with an AGC target of 2.0E5 and a maximum injection time of 50 ms. Dynamic exclusion was set for 20 seconds with a mass tolerance of +/−10 ppm. Isolation for MS2 scans was 1.6 Da using the quadrupole, and the most intense ions were sequenced using Top Speed for a 3 second cycle time. All MS2 sequencing was performed using higher energy collision dissociation (HCD) at 35% collision energy and scanned in the linear ion trap. An AGC target of 1.0E4 and 35 second maximum injection time was used. Raw files were searched against the UniProt human database using MaxQuant version 1.6.1.0 with Cysteine Carbamidomethylation as a fixed modification. Methionine oxidation and protein N-terminal acetylation were searched as variable modifications. All peptides and proteins were thresholded at a 1% false discovery rate (FDR).

### Immunofluorescence

Cells were plated in wells of Corning 96 well plates (#3603) the night before drug treatment. Cells were either treated with 100 nM, 1 µM, or 10 µM harmine/L41/INDY for 48 hours before receiving 4 Gy of irradiation. Cells were then rinsed twice with PBS and fixed with 4% formaldehyde for 20 minutes. Following fixation, antibodies for 53BP1 (Abcam, ab21083), γH2AX (Millipore), and Hoescht (Thermo Fisher Scientific) were used for fluorescent detection in fixed cells. The cells were then imaged with the Yokogawa Cell Voyager CV1000 confocal Scanner system using a 20x objective. All wells were imaged using a high throughput program in the CV1000 Acquisition software, allowing for 4 images per well to be taken with multiple confocal planes and computational autofocus. MIP files were then utilized for analysis by R-package Focinator v2^79^. In brief, the number of foci were counted within each nucleus, excluding nuclei on edge of each frame. Li thresholding was used, and noise/cutoff thresholds were set to 25 for both foci channels. Cells with a nucleus containing ten or more 53BP1 foci are considered 53BP1 + cells. This processing was done on 16 frames across 4 biological replicates in each treatment group, resulting in total cell counts between 400 and 1000. Count numbers were pooled across biological replicates for a weighted average and a two-proportion test was used between each drug treated condition/CRISPR KO and WT HeLa cells; represented by p-values outlined in Fig. 3 and Supplemental Fig. 2.

### Immunoblotting and immunoprecipitation

HeLa cells were lysed with RIPA buffer, sonicated using a Bioruptor Pico (Diagenode) for 10 cycles of 30 seconds on/30 seconds off, and centrifuged at 14,000 × g for 15 minutes. A Pierce BCA protein assay kit was used to determine protein concentrations and samples of 20 µg total protein were resolved using polyacrylamide gels and probed with the corresponding antibody for protein of interest. DYRK1A immunoprecipitations were done in triplicate using one of four antibodies: Abnova H00001859-M01, Abcam 69811, Bethyl A303-801A or Santa Cruz sc-12568. Antibodies were bound to bead- protein A/G mixtures overnight prior to affinity purification from protein lysate. Following a 15 min incubation with benzonase, protein lysates were precleared over protein A/G sepharose beads (GE Healthcare, Chicago, IL) mixture with no antibody for 1 hour. Beads were then spun down and cleared lysate was incubated with Antibody-bead mixture for 4 hours at 4 °C. Affinity purified proteins were eluted off beads using 0.1 M Glycine pH 2.75 twice for 30 minutes.

### Cell cycle analysis

10 µM BrdU was added to HeLa cells growing in culture for 60 minutes prior to trypsinizing and collection. Cells were washed with PBS and fixed using high grade ethanol at −20 °C. Cells were treated with 2 N HCl/Triton X-100 for 30 minutes, pelleted and resuspended in Borax to neutralize the sample. Cells were stained with a BrdU-FITC (BioLegend, San Diego California) antibody and propidium iodide (Sigma) for two-dimensional flow cytometry separation of DNA content. Cells were then separated into G0/G1, S, and G2/M phase based on gating of the two parameters.

### Clonogenic survival assay

Wild type or DYRK1A KO HeLa cells were plated at seeding density in T-25 flasks 24 hrs prior to irradiation with either 0, 4 or 8 Gy of x-ray. Cells were then immediately plated in 10 cm dishes at a predetermined number of cells (250 cells for 0 Gy, 500 cells for 4 Gy and 1000 cells for 8 Gy) and stored in an incubator for 10 days. Cells were then washed, fixed and stained with gentian violet. After drying plates overnight, plates were imaged, and colonies were counted using the clono-counter java package from Niyazi *et al*.^80^.

### 3xFlag WT FL_DYRK1A protein co-purification

HEK293s were transfected with 3x FLAG WT FL_DYRK1A vector using lipofectamine 2000CD. Cells were harvested 24 hrs after transfection and lysed with flag lysis buffer ((50 mM Tris pH 7.6, 0.15 M NaCl, 5% Glycerol, 0.1 M EDTA, 0.02% NP-40) with protease/phosphatase inhibitors and probe tip sonication. Lysate was applied to Anti-FLAG M2 affinity Gel (Sigma A2220) for overnight binding. Flag beads were washed with Tris salt buffers (50 mM Tris pH 7.6, 0.5/0.3/0.15 M NaCl), and eluted (50 mM Tris pH 7.6, 0.15 M NaCL, 150 ng/uL 3x FLAG peptide (Sigma F4799).

### Kaplan-Meier analysis

Kaplan-Meier analysis was done using KMplotter, a web-based analysis tool^74,75^ (http://kmplot.com/analysis/). An RNA-seq database was manually curated from publicly available gene expression data through GEO, EGA, and TCGA for samples with associated clinical data (patient outcome). Survival probability was evaluated by high or low DYRK1A mRNA expression based on an optimal expression cutoff calculated between the upper and lower quartiles for each cancer type.

## Supplementary information

Supplementary Figures 1-4

Supplemental Table 1

Supplemental Table 2

Supplemental Table 3

## Data Availability

The datasets generated during and/or analyzed during the current study are available in the MassIVE repository: ftp://massive.ucsd.edu/MSV000082881.
