# Supplementary material for: The nuclear interactome of DYRK1A reveals a functional role in DNA damage repair
Source: Sci Rep. 2019 Apr 25;9:6539. doi: 10.1038/s41598-019-42990-5 (PMC6483993; doi:10.1038/s41598-019-42990-5)
Supplement: Supplementary file 1 — Supplementary Figures 1-4 [file 41598_2019_42990_MOESM1_ESM.pdf]

The nuclear interactome of DYRK1A reveals a functional role in DNA damage repair

Steven E. Guard[1], Zachary C. Poss[1], Christopher C. Ebmeier[1], Maria Pagratis[1], Helen Simpson[1], Dylan J. Taatjes[2], William M. Old[1,3]*

[1] Department of Molecular, Cellular and Developmental Biology, University of Colorado, Boulder, CO

[2] Department of Biochemistry, University of Colorado, Boulder, CO

[3] Linda Crnic Institute for Down Syndrome, University of Colorado School of Medicine, Anschutz Medical Campus, Aurora, CO

*Corresponding Author: William M. Old, Molecular, Cellular and Developmental Biology, University of Colorado Boulder, Boulder, CO 80309; T: 303.492.9551; E: William.Old@colorado.edu

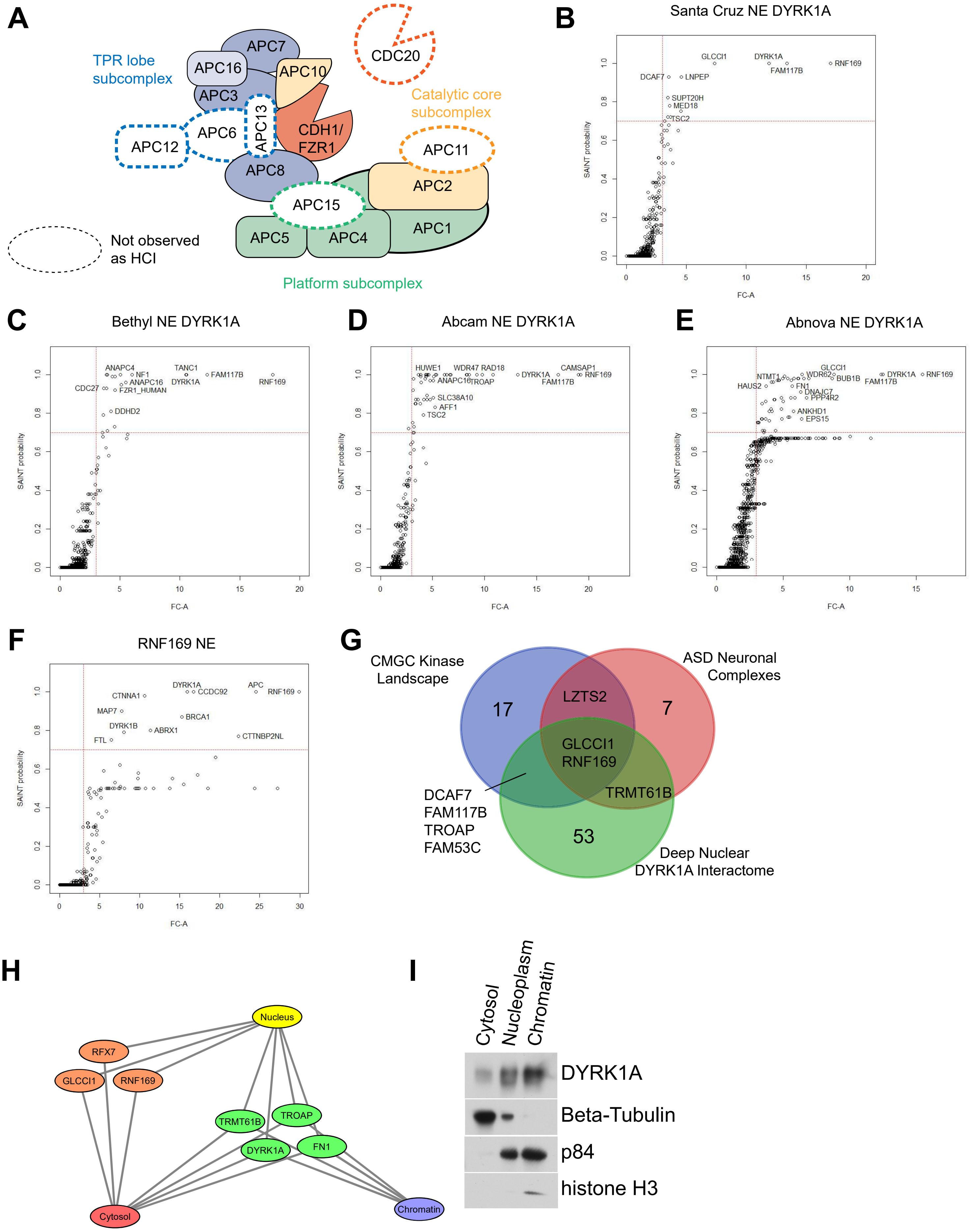

Supplemental Figure 1

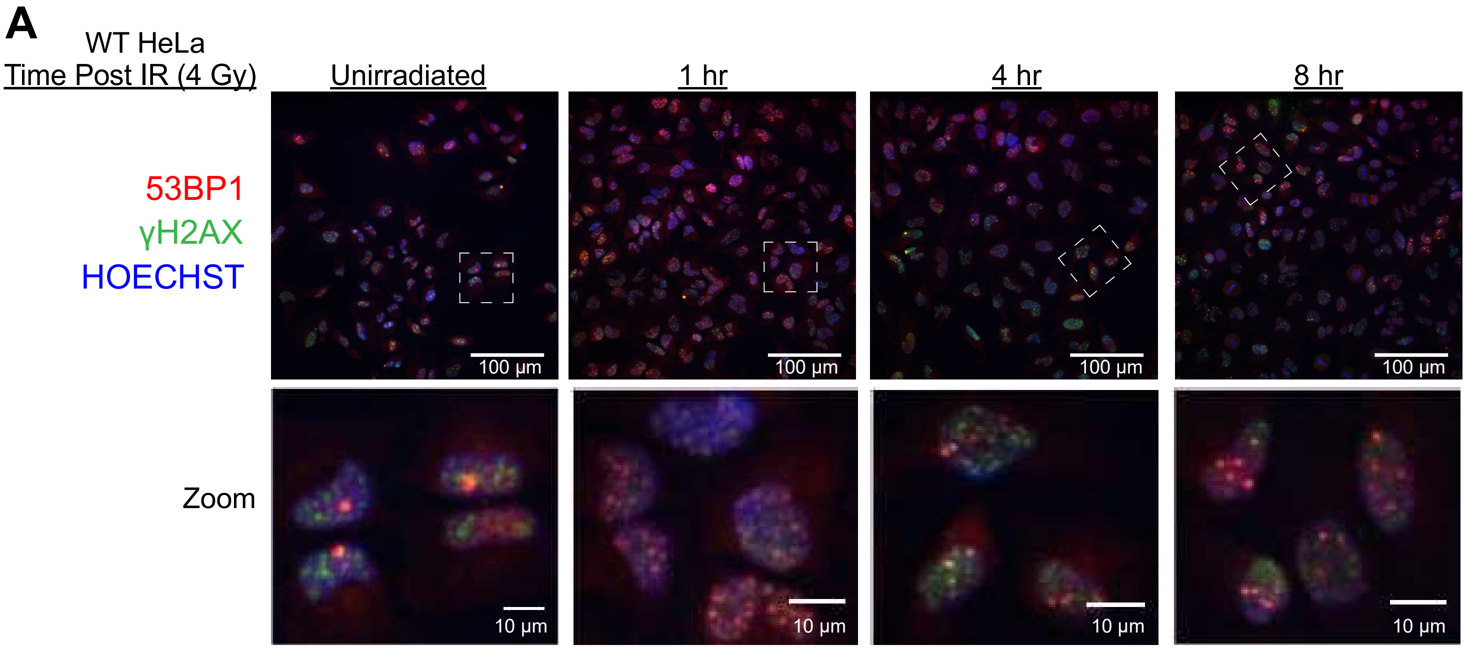

% of 53BP1+ cells	0%	52%	16%	6%
% of γH2AX+ cells	26%	37%	47%	38%
# of cells	683	680	462	495

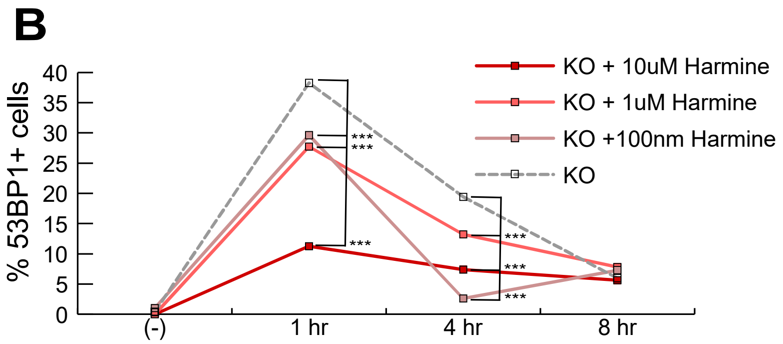

Supplemental Figure 2

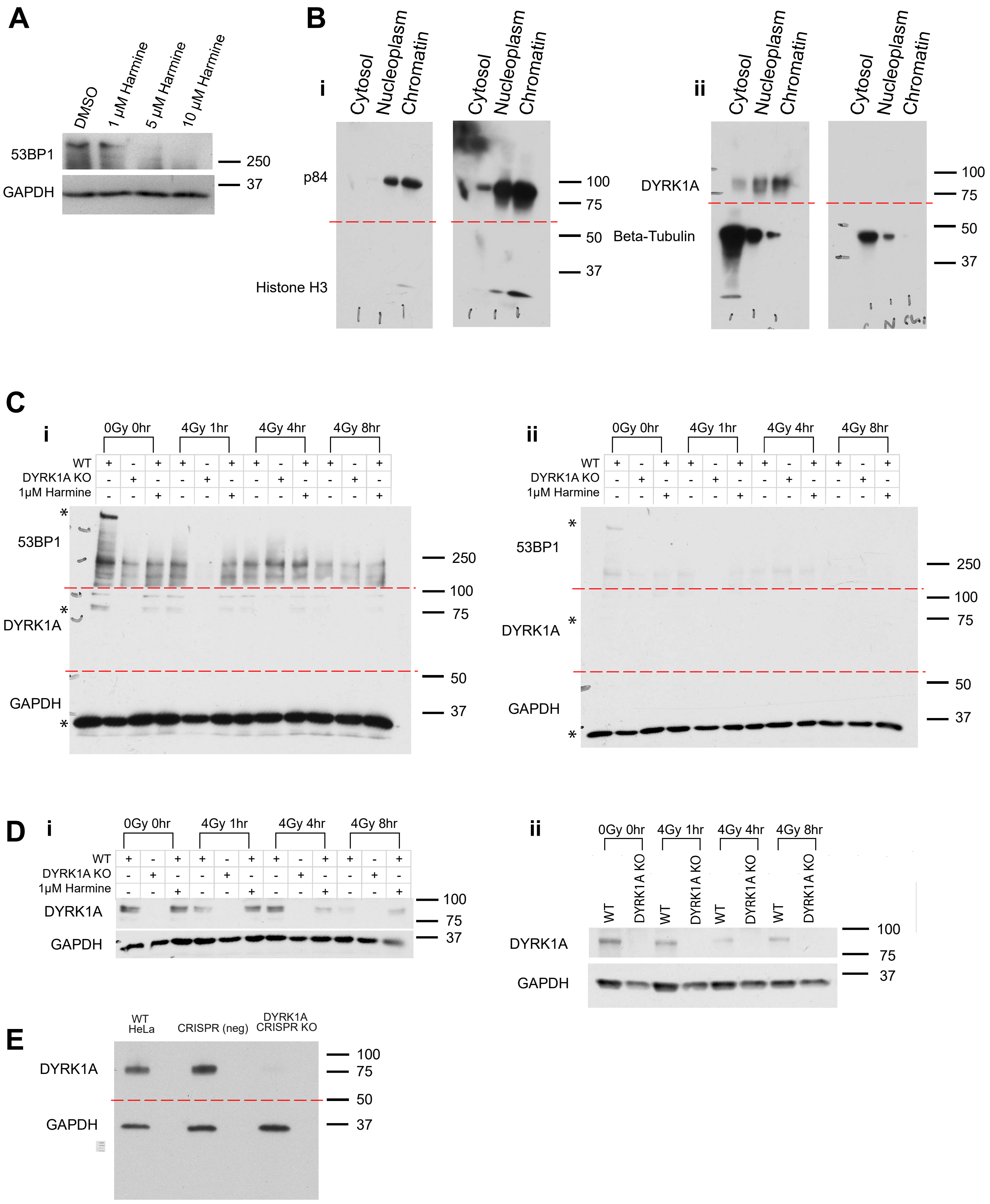

Supplemental Figure 3

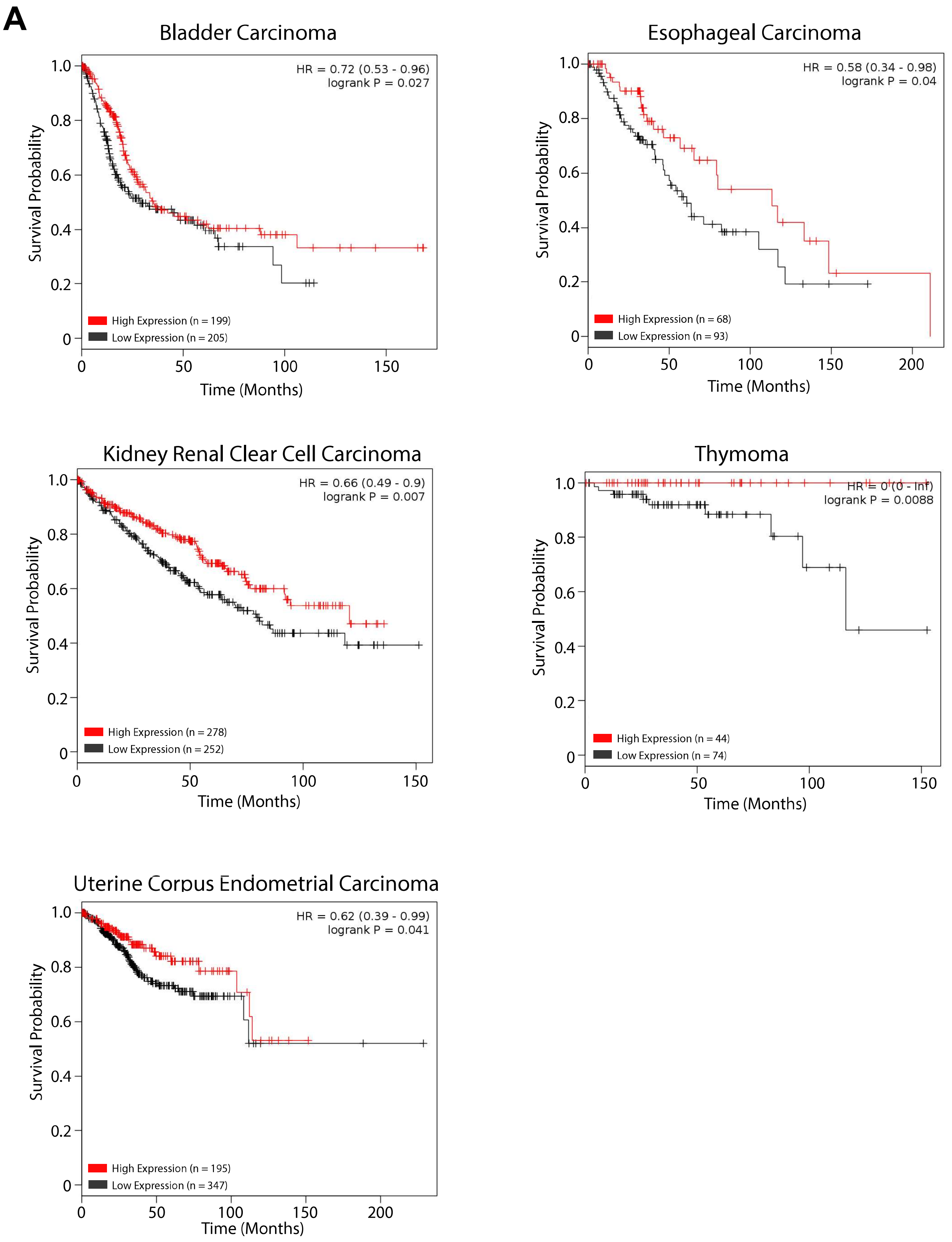

Supplemental Figure 4

Supplemental Figure 1: Statistical thresholding of nuclear affinity purification networks. **(A)** Graphical representation of anaphase promoting complex subunits identified as HCIs in the DYRK1A nuclear interactome. **(B-F)** Scatter visualization of HCI cutoffs on total protein appearance in affinity purifications. Each plot represents FC-A and SAINT probability calculated on triplicate biological replicates (NE = nuclear extract) **(G)** Comparison of DYRK1A interactomes with literature reported interaction sets. Green: Nuclear DYRK1A HCI interactome; Blue: Varjosalo *et al.*[30]; Red: Li *et al.* (2015)[59] **(H)** DYRK1A was immunoprecipitated using the Abnova antibody in triplicate from the cytosolic fraction, nuclear fraction and nuclear pellet (chromatin bound fraction) of HeLa cells. Nodes in network represents only shared HCI's between compartments. Edges represent which subcellular fraction an HCI was found in. **(I)** Immunoblot for subcellular fractionation markers from 20 µg of loaded protein from each subcellular fraction. P84 and Histone H3 bands were cropped from portions of the same blot. DYRK1A and ß-Tubulin bands were cropped from a separate second blot. (Full molecular weight range seen in Suppl. Fig S3B.)

Supplemental Figure 2: Representative images of 53BP1 recruitment profile in HeLa cells, and cell cycle influence of DYRK1A KO. **(A)** N ≈ 400- 1000 cells per condition. Blue: Hoechst; Green: γH2AX; Red: 53BP1. γH2AX+ cells ≥ 10 foci/ cell; 53BP1+ cells ≥ 10 foci/ cell. (53BP1 noise cut off: 15; γH2AX noise cut off 20) **(B)** DYRK1A KO cells were treated with 100 nM, 1 µM or 10 µM harmine 48 hours prior to IR treatment. Cells were fixed and quantified as in Fig. 3A. (Proportion test: drug treated cells vs DYRK1A KO; *: $p < 0.05$; **: $p < 0.01$; ***: $p < 0.001$).

Supplemental Figure 3: Additional western blot replicates and supporting data. Ladder values are given in kDa. **(A)** Loss of 53BP1 expression by 3 hours post harmine treatment in SH-SY5Y cells **(B)** Immunoblots for subcellular fractionation markers from 20 µg of loaded protein from each subcellular fraction (given at two different exposures to provide appropriate band intensity). **(i)** Blot was cut at 50kDa and probed for p84 above 50kDa and Histone H3 below 50kDa. **(ii)** Blot was cut slightly at approximately 65 kDa and probed for DYRK1A above 65 kDa and ß-Tubulin below 65 kDa. **(C)** Immunoblot for 53BP1, DYRK1A and GAPDH expression in either WT, DYRK1A KO or 1 µM harmine treated WT cells at 0 hrs, 1 hr, 4 hrs and 8 hrs following 4 Gy of IR. Blot was cut at 100kDa and 50kDa before probing for 53BP1 above 100 kDa, DYRK1A from 50- 100 kDa and GADPH below 50 kDa. **(i)/(ii)** Two different exposures of the same blot with x-ray film. (**D)** Replicates of DYRK1A down regulation in WT HeLa cells post IR. **(i)** repeat conditions of Figure 4A. **(ii)** Third replicate of DYRK1A degradation in WT vs DYRK1A Ko cells post IR **(E)** Immunoblot for DYRK1A and GAPDH loading control expression

in WT and DYRK1A KO cells. Blot was cut at 50 kDa and probed for DYK1A above 50kDa and GADPH below 50kDa**.**

Supplemental Figure 4: High DYRK1A expression correlates with high survival probability in multiple cancers. (A) Kaplan-Meier survival analysis of tumors representing five different cancer types. Patients with high DYRK1A expression in their tumors had increased survival probability over those with low DYRK1A expression. Plots were generated with KM plotter[74,75].

Supplementary Files:

Supplementary Table 1. Table of antibody registry information for DYRK1A antibodies used in immunoprecipitation experiments

Supplementary Table 2. DYRK1A nuclear interactions identified in this study and collected from literature

Supplementary Table 3. Table of fluorescent foci counts
